# Al(OH)_3_ facilitated synthesis of water-soluble, magnetic, radiolabelled and fluorescent hydroxyapatite nanoparticles†
†Electronic supplementary information (ESI) available: Conjugation of NPs with dyes, radiolabelling for NPs, NMR spectra, XRD, IR, zeta potential, DLS size distribution, TEM images and TGA data of NPs, fluorescent images of NPs. See DOI: 10.1039/c5cc02259b



**DOI:** 10.1039/c5cc02259b

**Published:** 2015-05-11

**Authors:** X. Cui, M. A. Green, P. J. Blower, D. Zhou, Y. Yan, W. Zhang, K. Djanashvili, D. Mathe, D. S. Veres, K. Szigeti

**Affiliations:** a King's College London , Division of Imaging Sciences and Biomedical Engineering , 4th Floor of Lambeth wing , St Thomas Hospital , London SE1 7EH , UK . Email: philip.blower@kcl.ac.uk ; Email: mark.a.green@kcl.ac.uk; b King's College London , Department of Physics , Strand Campus , London , WC2R 2LS , UK; c Department of Mathematical Science , Loughborough University , Loughborough , LE11 3TU , UK; d School of Chemistry , Nottingham University , Nottingham , NG7 2RD , UK; e Department of Biotechnology , Delft University of Technology , Julianalaan, 136 , 2628 BL , Delft , The Netherlands; f CROmed Ltd , Baross u. 91-95 , H-1047 , Budapest , Hungary; g Department of Biophysics and Radiation Biology , Semmelweis University , IX, Tüzoltó u. 37-47 , H1094 , Budapest , Hungary

## Abstract

Magnetic and fluorescent hydroxyapatite nanoparticles were synthesised using Al(OH)_3_-stabilised MnFe_2_O_4_ or Fe_3_O_4_ nanoparticles as precursors. They were readily and efficiently radiolabelled with ^18^F. Bisphosphonate polyethylene glycol polymers were utilised to endow the nanoparticles with excellent colloidal stability in water and to incorporate cyclam for high affinity labelling with ^64^Cu.

## Abstract 2

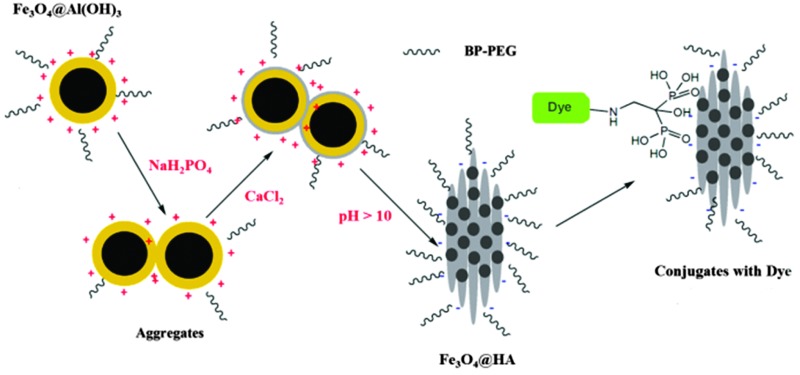
Radiolabelling, magnetic and fluorescent properties are incorporated in one single Fe_3_O_4_@HA nanoparticle with potential application as trimodal probes.

Molecular imaging techniques, including magnetic resonance imaging (MRI), positron emission tomography (PET), single photon emission computed tomography (SPECT) and fluorescence optical imaging, play an increasingly important role in clinical diagnosis and management of disease, as well as medical and biological research. Multimodal imaging recently has gained attention because of its potential to overcome the limitations of individual imaging modalities and to provide more accurate and complete physiological information at sites of disease.^1–3^ Numerous nanoparticles (NPs) have been studied as multimodal imaging contrast agents due to their multi-functionality and potential for surface modification.^4–7^ An adequate multimodal nanoparticulate contrast agent must be multifunctional, biocompatible and colloidally stable. The NPs should be uniform in morphology and size, so that they share similar *in vivo* behaviour, and chemically stable to ensure that the signal of each modality reflects the same anatomic position.

Hydroxyapatite (HA) has attracted much interest as the basis of multifunctional probes very recently,^8–11^ because of its biocompatibility and high affinity for fluoride which allows facile labelling with the positron emitter ^18^F. Fluorescent HA can be obtained by either doping with rare earth cations^8,11^ or by conjugation with organic dyes.^10^ HA is not an ideal fluorescent host matrix, so luminescent rare earth doped HA nanocrystal requires up to 20% replacement of OH^–^ by F^–^ (maximum theoretical value for fluoride substitution), to minimise the quenching of the excited sate of rare earth cations.^8,12,13^ As a result, such HA is no longer suitable for ^18^F radiolabelling. It has been reported that magnetic iron oxide NPs can be deposited on the surface of HA aggregates or NPs *via* thermolysis^11^ or a wet chemistry approach.^9^ One problem that remained unsolved for both synthetic approaches is how to effectively isolate the desired Fe_3_O_4_–HA composites from the unwanted iron oxides and HA nanoparticles. Moreover, all these multifunctional HA NPs suffer from the problem of aggregation or large size to some extent, which is an obstacle for their biological or medical applications. In this work, we present a novel synthesis of magnetic and fluorescent HA nanocomposites with uniform size and morphology, and excellent colloidal stability in water by using Fe_3_O_4_ nanoparticles stabilised with Al(OH)_3_ as a template. The radiolabelling, magnetic and optical properties were investigated, to demonstrate potential for application as tri-modal probes for MR, PET and optical imaging.

Our strategy is to synthesise HA using water-soluble magnetic Fe_3_O_4_@Al(OH)_3_ or MnFe_2_O_4_@Al(OH)_3_ NPs as templates. The advantages of this approach is the small hydrodynamic size of the template particles and their excellent colloidal stability, provided by the Al(OH)_3_ layer as reported previously.^14^ More importantly, the layer of Al(OH)_3_ can be readily removed as it is soluble under basic pH conditions. The design incorporated bisphosphonate polyethylene glycol (BP-PEG) polymers (Scheme 1) to stabilise NPs after the formation of HA on the surface, to take advantage of the outstanding binding affinity of bisphosphonates to HA.

**Scheme 1:**
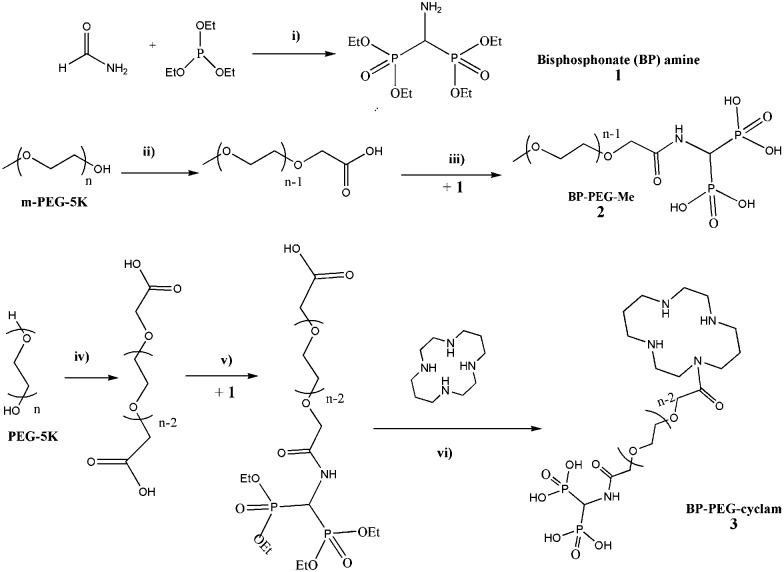
Synthesis of bisphosphonate PEG, (i) dropwise addition of POCl_3_ at –7.5 °C under N_2_, extract with CH_2_Cl_2_/H_2_O/CH_2_Cl_2_ under basic/acidic/basic conditions; (ii) CrO_3_/H_2_SO_4_, 24 hours; (iii) react with **1** and *N*,*N*′-dicyclohexylcarbodiimide (DCC), CH_2_Cl_2_, 18 h then with TMBS, remove solvent, stir in methanol; (iv) CrO_3_/H_2_SO_4_, 24 hours; (v) react with **1** and DCC, CH_2_Cl_2_, 18 hours; (vi) react with 1,4,8,11-tetraazacyclotetradecane and DCC, CH_2_Cl_2_, 18 hours, then with TMBS, remove solvent, stir in methanol for 2 h.

Bisphosphonate amine **1** was obtained *via* a slightly modified version of the previously reported protocol.^15^ PEG carboxylic acids were obtained by oxidation of corresponding PEG polymers with CrO_3_/H_2_SO_4_ *via* the reported protocol.^16^ The bisphosphonate (BP) or 1,4,8,11-tetraazacyclotetradecane (cyclam) were grafted to PEG polymeric chain *via* amide formation mediated by *N,N*′-dicyclohexylcarbodiimide (DCC), followed by deprotection with bromotrimethylsilane (TMBS).^17^ BP-PEG-Me **2** and BP-PEG-cyclam **3** were purified by dialysis for over 24 hours using a membrane with a cut-off size of 3500 Da to remove unconjugated small molecules such as bisphosphonate amine **1** and 1,4,8,11-tetraazacyclotetradecane. The conjugation of bisphosphonate and PEG was confirmed by the change in chemical shift in the ^31^P NMR spectrum (from 20 ppm for free bisphosphonate to 12.8 ppm for BP-PEG, see ESI†).

Nanoparticulate precursors MnFe_2_O_4_@Al(OH)_3_ and Fe_3_O_4_@Al(OH)_3_ were obtained *via* a method reported by our group previously.^14^ Typically, 4 ml Fe_3_O_4_@Al(OH)_3_ colloids (concentration of Fe_3_O_4_, *ca.* 8 mg ml^–1^) and 200 mg BP-PEG-Me polymers **2** were placed in a 500 ml flask containing 300 ml water. Under stirring, 4 ml 0.1 mol l^–1^ NaH_2_PO_4_ and 4 ml 0.2 mol l^–1^ CaCl_2_ aqueous solutions were added sequentially. This light brown solution was refluxed overnight after the addition of 30 ml 28% ammonia water. The NPs were collected by centrifugation at 2000 g for 30 minutes, re-dissolved in 10 ml water and freeze dried.

The X-ray powder diffraction (XRD) pattern of NPs indicates co-existence of HA and Fe_3_O_4_ (or MnFe_2_O_4_). TEM images show an olive-like morphology for Fe_3_O_4_@HA and MnFe_2_O_4_@HA NPs, which is significantly different both in size and in morphology from pure HA NPs synthesised under the same conditions (Fig. 1). Particle analysis by TEM gave a mean size of 60.3 nm (major axis) × 29.7 nm (minor axis) for Fe_3_O_4_@HA NPs (Fig. 1c and d). MnFe_2_O_4_@HA NPs displayed a similar aspect ratio to Fe_3_O_4_@HA NPs, but the size was almost doubled. These results indicate an important role of BP-PEG-Me in reducing particle size.

**Fig. 1:**
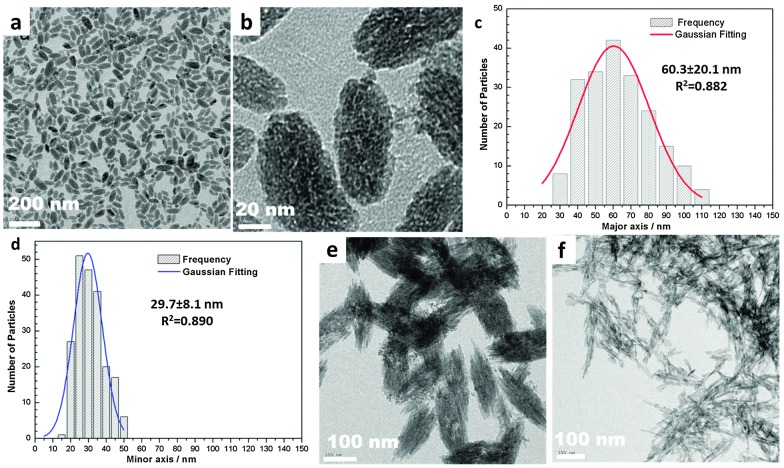
TEM images (a) and (b) and size distribution (c) and (d) (major and minor axis, respectively) of Fe_3_O_4_@HA NPs synthesised from Fe_3_O_4_@Al(OH)_3_ in the presence of BP-PEG-Me; (e) TEM image of MnFe_2_O_4_@HA NPs synthesised in absence of BP-PEG-Me **2**; (f) TEM image of pure HA NPs.

The hydrodynamic size of NPs during the synthesis was monitored by dynamic light scattering (DLS) experiments. No obvious change was observed after adding the solutions of NaH_2_PO_4_ and CaCl_2_ into the solution of Fe_3_O_4_@Al(OH)_3_ NPs, and it remained around 80 nm. This leads to a hypothesis that phosphate anions adsorb on the highly positive charged surface of Fe_3_O_4_@Al(OH)_3_ NP and then react with the subsequently added Ca^2+^ to form calcium phosphate around the NPs. At the elevated temperature and basic solution environment, the outer layer of calcium phosphate was converted to crystallised HA, meanwhile the Al(OH)_3_ was dissolved, resulting in the formation of Fe_3_O_4_@HA NPs. The diminished peak due to –OH around 3300–3500 cm^–1^ in the IR spectrum, together with XRD patterns, confirm the replacement of Al(OH)_3_ layer by HA. The changes in surface potential of NPs could not be monitored by measuring the zeta potential during the process, since the polymeric PEG imposes a thick hydration layer on NP surface and zeta potential no longer correlates to the surface potential. Therefore the surface potential was monitored in the absence of BP-PEG-Me, and a significant decrease in zeta potential was observed after the addition of NaH_2_PO_4_ solution, from 42.5 mV to 24.9 mV. This is presumed to be due to the adsorption of phosphate anions on the surface, since the changes in pH should be negligible in this case. The zeta potential slightly increased back to 27.1 mV after adding the CaCl_2_ solution, indicating a reaction of calcium cations and phosphate anions. Similar results were also observed for MnFe_2_O_4_@HA NPs. In this synthesis approach, the positively charged Al(OH)_3_ layer is essential for the formation of MnFe_2_O_4_@HA or Fe_3_O_4_@HA. Using the MnFe_2_O_4_ NPs colloids instead of MnFe_2_O_4_@Al(OH)_3_ as precursors, a simple mixture of magnetic MnFe_2_O_4_ and non-magnetic HA NPs was obtained, identifiable as two kinds of NPs with apparently different morphology and size on TEM images.

Both MnFe_2_O_4_@HA and Fe_3_O_4_@HA NPs were coated by BP-PEG-Me polymers **2** during the synthesis, as confirmed by *ca.* 18% mass loss on thermogravimetric analysis (TGA). Due to the strong interactions between the bisphosphonate group of **2** and MnFe_2_O_4_@HA and Fe_3_O_4_@HA NPs,^17,18^ both NPs exhibit long-term colloidal stability in aqueous solution, even in high ionic strength environment such as PBS. The hydrodynamic size of Fe_3_O_4_@HA and MnFe_2_O_4_@HA NPs remained at 50.7 nm and 60.3 nm, respectively, for over two months (Fig. 2a). The excellent colloidal stability and small hydrodynamic size of MnFe_2_O_4_@HA and Fe_3_O_4_@HA make them potentially suitable for biological or medical applications.

**Fig. 2:**
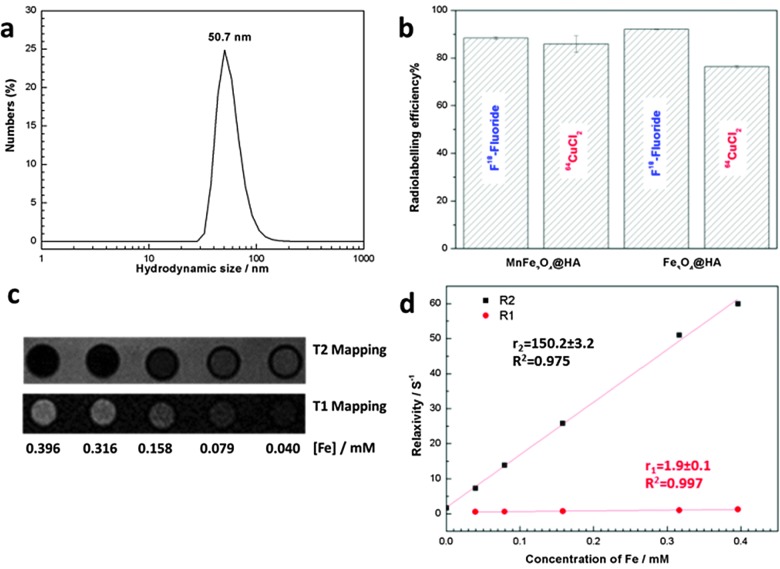
(a) DLS size distribution of 1 mg ml^–1^ Fe_3_O_4_@HA NPs in aqueous solution; (b) radiolabelling of MnFe_2_O_4_@HA and Fe_3_O_4_@HA NPs with ^18^F-fluoride and ^64^CuCl_2_ (the latter after sonicating the particles with BP-PEG-cyclam); (c) T_1_ and T_2_ weighted MR images of the solution containing Fe_3_O_4_@HA NPs, and (d) relaxivities of Fe_3_O_4_@HA NPs. Concentration of iron in the solution was measured by ICP-MS.

Unsurprisingly, because of the high affinity of fluoride for HA,^19^ both NPs exhibit a high radiolabelling efficiency with ^18^F-fluoride, up to 88.3 ± 0.5% for 0.3 mg MnFe_2_O_4_@HA NPs and 92.1 ± 0.1% for 0.3 mg Fe_3_O_4_@NaYF_4_ NPs (Fig. 2b). Labelling and purification was readily achieved in less than 23 min. To provide a means of incorporating the positron emitter ^64^Cu, the NPs were sonicated in 1 mg ml^–1^ BP-PEG-cyclam solution for 30 minutes to allow replacement of a fraction of BP-PEG-Me by BP-PEG-cyclam, and free BP-PEG-polymers were removed by centrifugation before mixing with radioactivity. The resulting particles showed a high ^64^Cu radiolabelling efficiency in a short time (<5 minutes) (Fig. 2b). Both NPs display essentially the magnetic properties of Fe_3_O_4_ or MnFe_2_O_4_ NPs and are active on MR images (Fig. 2c and d). The transverse (*r*_2_) and longitudinal (*r*_1_) relaxivities of Fe_3_O_4_@HA NPs were measured to be 150.2 ± 3.2 mM^–1^ s^–1^ and 1.9 ± 0.1 mM^–1^ s^–1^, respectively, at 3 T magnetic field. As expected, the relaxivities of NPs could be improved by altering the ratio of magnetic component and non-magnetic HA, since *r*_2_ is proportional to the volume fraction of magnetic component.^20^ For example, the *r*_2_ of MnFe_2_O_4_@HA NPs could be dramatically improved from 105.7 ± 3.5 mM^–1^ s^–1^ to 246.5 ± 15.9 mM^–1^ s^–1^ by doubling the amount of MnFe_2_O_4_@Al(OH)_3_ while keeping the amount of NaH_2_PO_4_ and CaCl_2_ solutions the same during the synthesis. High transverse relaxivity of these magnetic hydroxyapatite NPs as well as a high ratio of *r*_2_/*r*_1_ demonstrate their potential application as T_2_ contrast agents on MR imaging.

Fluorescent HA is normally produced either by doping with rare earth cations (Eu or Tb),^8,11,21^ or by conjugation with organic dyes.^10,22^ Here we conjugated the fluorescent dyes Maria blue and Alexa Fluor® covalently to the NPs surface using sodium pamidronate as an aminobisphosphonate linker (see ESI†). The amine group of pamidronate is reactive for NHS ester dyes to form stable amide bonds while its bisphosphonate group interacts strongly with the Ca or Fe at the surface of NPs; dyes are thus linked to NPs without the risk of leakage. Fluorescent spectra in Fig. 3 show an emission at 455 nm for the conjugates of Fe_3_O_4_@HA and Maria blue under excitation at 365 nm, and an emission at 517 nm for the Alexa Fluor® 488 conjugates under excitation at 488 nm. More importantly, the fluorescence of these conjugated NPs is stable and strong even after being stored at room temperature for over one month, implying the potential applications as optical contrast.

**Fig. 3:**
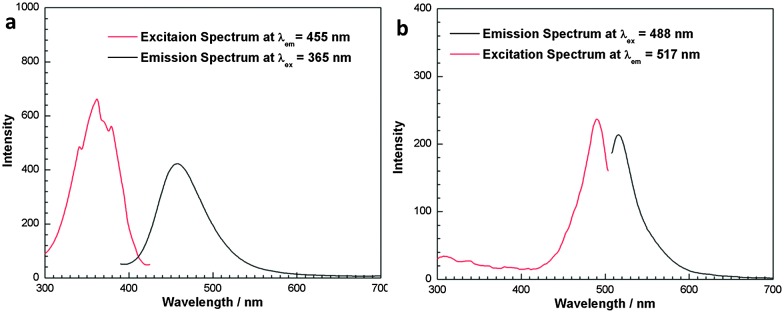
Emission and excitation spectra of aqueous solutions of Fe_3_O_4_@HA conjugates with (a) Maria blue and (b) Alexa Fluor® 488.

In summary, we have presented a facile approach to synthesise magnetic and fluorescent hydroxyapatite nanoparticles with a well-defined morphology and uniform size, using Al(OH)_3_-stabilised Fe_3_O_4_ or MnFe_2_O_4_ NPs as templates. The change from the highly positively charged surface and base-solubility of the Al(OH)_3_ layer to the neutral or slightly negative zeta potential and acid solubility of the Fe_3_O_4_@HA particles is likely to offer alternative biological properties. These NPs are promising candidates for development as tri-modal probes for MR, PET and optical imaging, since they display excellent colloidal stability and high radiolabelling efficiency both for ^64^Cu and for ^18^F, as well as fluorescent and magnetic properties. Radiolabelling with other metallic radioisotopes will also be achievable by replacing cyclam with corresponding chelators. This synthesis approach allows us to tune the magnetic properties of particles by altering the ratio of precursors, without decreasing the radiolabelling efficiency or fluorescent property. The flexible conjugation method ensures that dyes with different wavelengths could be selected for different applications. The synthesis strategy for conjugation of BP-PEG-cyclam can also be applied to the conjugation of BP-PEG with peptides or antibodies, leading to the application in targeted imaging.

We acknowledge Mr Dirk Krüger from Division of Imaging Science and Biomedical engineering, King's College London, for his works on the magnetic relaxivitity measurement. This research was supported by the Centre of Excellence in Medical Engineering Centre funded by the Wellcome Trust and EPSRC under grant number WT088641/Z/09/Z, and the Kings College London and UCL Comprehensive Cancer Imaging Centre funded by the CRUK and EPSRC in association with the MRC and DoH (England), and by the National Institute for Health Research (NIHR) Biomedical Research Centre at Guy's and St Thomas' NHS Foundation Trust and King's College London. The views expressed are those of the author(s) and not necessarily those of the NHS, the NIHR or the Department of Health.
