# Supplementary material for: Al(OH)3 facilitated synthesis of water-soluble, magnetic, radiolabelled and fluorescent hydroxyapatite nanoparticles
Source: Chem Commun (Camb). 2015 May 11;51(45):9332–5. doi: 10.1039/c5cc02259b (PMC4601318; doi:10.1039/c5cc02259b)
Supplement: Supplementary file 1 [file CC-051-C5CC02259B-s001.pdf]

Electronic Supplementary Material (ESI) for ChemComm.
This journal is © The Royal Society of Chemistry 2015

Supporting Information

$Al(OH)_3$ Facilitated Synthesis of Water-soluble, Magnetic, Radiolabelled and Fluorescent Hydroxyapatite Nanoparticles

X. J. Cui,[ξ a] M. A. Green,[* a, b] P. J. Blower,[* a] D. W. Zhou,[c] Y. Yan,[d] W. Zhang,[e] K. Djanashvili,[e] D. Mathe,[f] D. S. Veres,[g] and K. Szigeti [g]

[a] Division of Imaging Science, 4th Floor of Lambeth wing, St Thomas Hospital campus, King's College London, SE1 7EH, London, UK

[b] Department of physics, Strand campus, King's College London, WC2R 2LS, London, UK

[c] Department of Mathematical Science, Loughborough University, Loughborough, LE11 3TU, UK

[d] School of Chemistry, University of Nottingham, Nottingham, NG7 2RD, UK

[e] Department of Biotechnology, Delft University of Technology, Julianalaan, 136, 2628 BL, Delft, the Netherlands

[f] CROmed Ltd, Baross u. 91-95, H-1047, Budapest, Hungary

[g] Department of Biophysics and radiation, Biology, Semmelweis University, IX, Tüzoltó u. 37-47, H0194, Budapest, Hungary

[*] Corresponding authors: Prof. Philip J. Blower (philip.blower@kcl.ac.uk) and Prof. Mark A. Green, (mark.a.green@kcl.ac.uk)

[ξ] Present address: Department of Materials, South Kensington campus, Imperial College London, SW7 2AZ

Experimental section

All chemicals are used as purchased without further purification. Water was obtained from an ELGA PureLabOptionQ system. ^{18}F and ^{64}Cu were produced using the PET Imaging Centre cyclotron at St

Thomas's Hospital, London, UK. ^{99m}Tc-MDP was obtained from the Nuclear Medicine Department, Guy's and St Thomas' Foundation Trust. Thermogravimetric analysis (TGA) was performed under a flow of nitrogen (20 ml/min) with a heating rate of 5 °C/min using a TA SDT-600 thermogravimetric analyser. XRD were recorded at room temperature on a PANalyticalX'Pert PRO diffractometer using Cu-$K\alpha_1$radiation (λ = 1.540598 Å) at 40 kV, 40 mA, at a scan speed of 0.02°/s and a step size of 0.026° in 2θ. TEM images were taken on Tecnai FEI T20 at Centre for Ultrastructural Imaging, King's College London. Particle analysis on TEM images were carried out byMatLab.DLS experiments were carried out on ZetasizerNano ZS from Malvern Instruments with a measure angle 175° and a 632.8 nm laser. Zeta potential for all samples was measured in neutral aqueous solution with a pH value ≈7.MR imaging was performed with a standard extremity flex coil on a clinical 3T Philips Achieva MRI scanner (Philips Healthcare, Best, The Netherlands).[1, 2] Proton and phosphorus NMR spectra for all samples were recorded in D_2O on Agilent-400 NMR spectrometer (400M Hz) at Delft University of Technology. Fluorescent images were recorded on CellVizio (MaunaKea Technologies, Paris, France) confocal laser fibre-optic endomicroscope at CROmed Ltd, Budapest, Hungary.

1. **Conjugation of NPs and Dyes.** Sodium pamidronate was synthesised on base of a previously reported procedure in literature[3]. The conjugation of organic dyes with sodium pamidronate was carried out by mixing a freshly prepared DMSO solution of dye (Maria blue or Alexa Fluor) and aqueous solution of sodium pamidronate in a $NaBF_4$ buffer (0.1 mol/l, pH=8.5). The resultant mixture solution was incubated at room temperature overnight, and stored in the dark for further conjugation with NPs. Typically, to a solution of 2 mg PEGylated Fe_3O_4@HA NPs in 20 ml, 100 μl solution containing Alexa Fluor® 488 NHS ester (20 μg, 0.03 μmol) and sodium pamidronate (40 μg, 0.16 μmol) was added under sonication. The mixture solution was kept at room temperature for over weekend, and then centrifuged at speed of 16000 rpm for 30 minutes to remove free dyes in the solution. The supernatant was discarded and the black residue was re-dissolved in water. Further purified was carried out by dialysis using a membrane with a cut-off-molecular size of 3500.

2. **Radiolabelling of NPs with ^{18}F-Fluoride, and $^{64}CuCl_2$**

All radiolabelling experiments in this work were carried out in triplicate, using 300 μl 1 mg/ml NPs solution. Typically, 200 μl aqueous [^{18}F] sodium fluoride solution containing *ca* 20 MBq radioactivity was added to a 1 ml centrifuge tube containing 300 μl NPs solution. After incubation at room temperature for 5 minutes, the radiolabeled NPs were isolated by centrifugation at 14000 rpm (Eppendorf centrifuge 5424) for 15 minutes. The radioactivity remaining in supernatant and on the NPs were measured separately by a CRC-25R dose calibrator (Capintec, USA), to yield a radiolabelling efficiency. The similar labelling method was used for ^{64}Cu. More details are available in previously reported work by our group,[1, 4, 5] but using BEP-PEG-cyclam as chelating ligands instead of small molecularbis(dithiocarmabate) bisphosphonate.

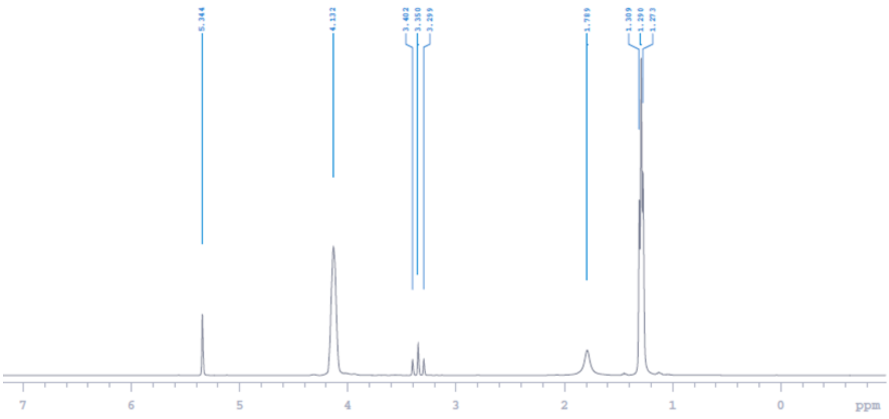

Figure S1 ^{1}H NMR spectrum ofbisphosphonate amine (**1**)

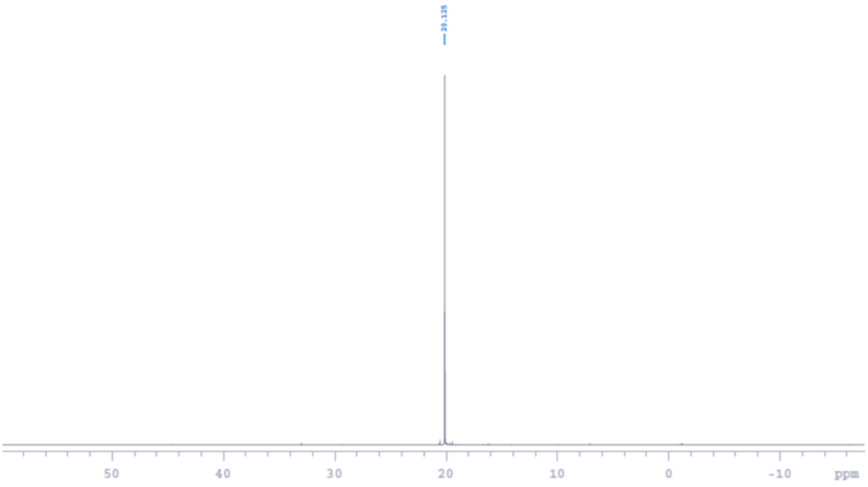

Figure S3. ^{31}P NMR spectrum of bisphosphonate amine (**1**)

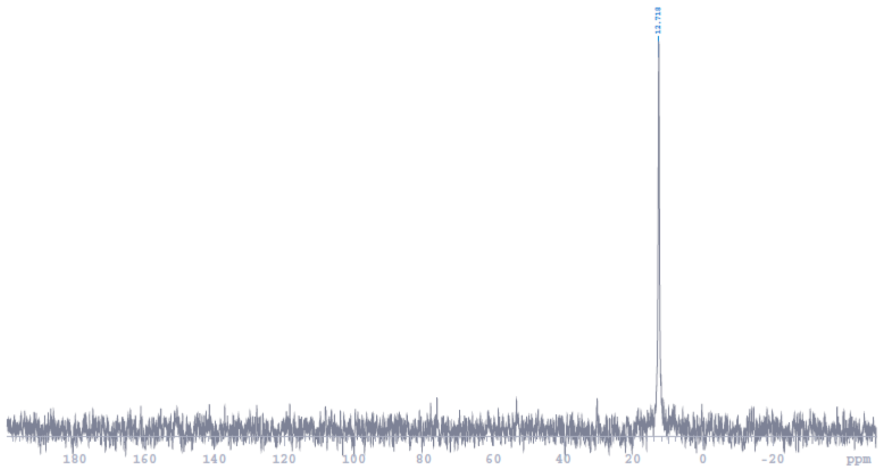

Figure S4. ^{31}P NMR spectrum of bisphosphonate PEG methyl ether (BP-PEG-CH_3, **2**)

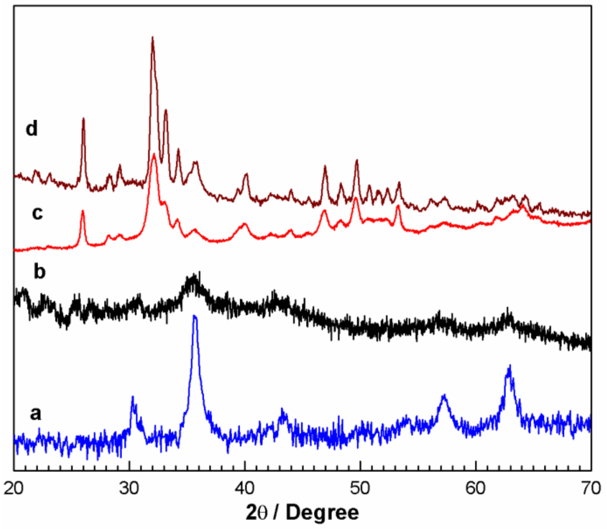

Figure S5. X-ray powder diffraction (XRD) patterns of NPs: **a**) $MnFe_2O_4$, **b**) $MnFe_2O_4$@$Al(OH)_3$, **c**) $MnFe_2O_4$@HA, and **d**) pure HA

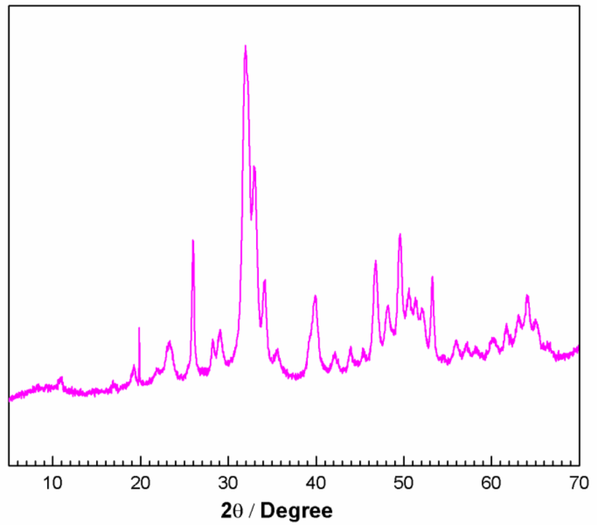

Figure S6. XRD pattern of Fe_3O_4@HA-BP-PEG-Me NPs

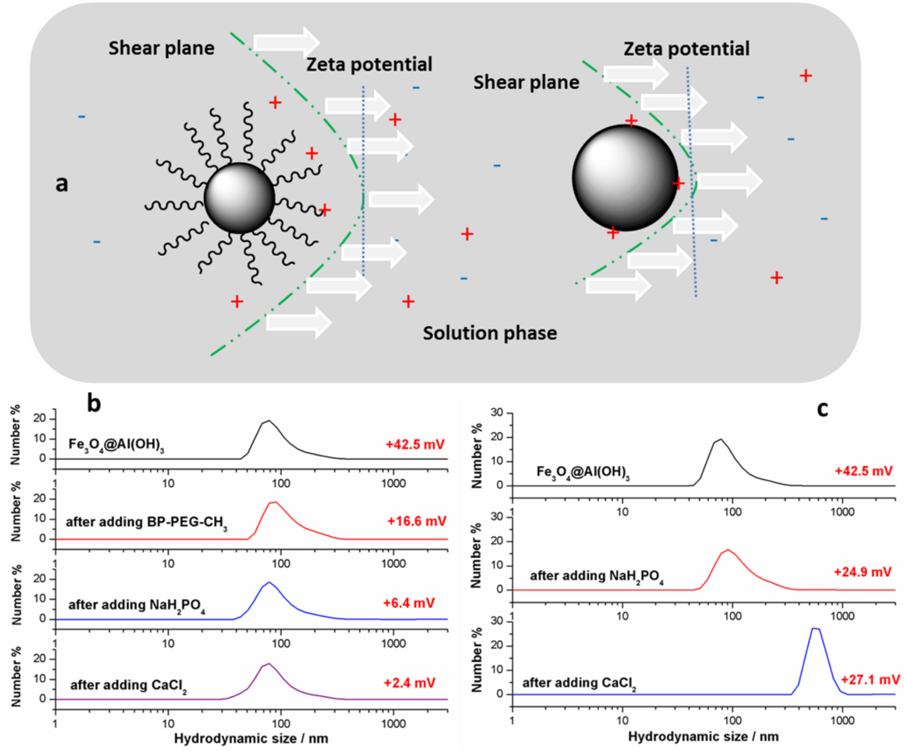

Figure S7. a) Schematic diagram of the coated and uncoated particle moving in the solution phase. **b**) the evolution of the zeta potential and hydrodynamic size of NPs in presence of BP-PEG, and **c**) the evolution of the zeta potential and hydrodynamic size of NPs in absence of BP-PEG

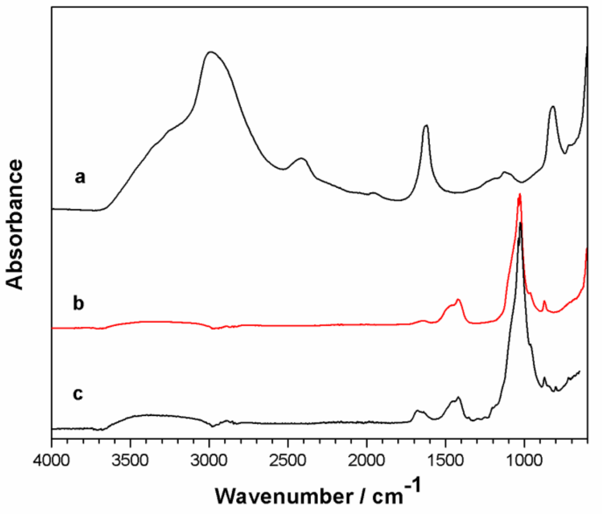

Figure S8. IR spectrum of **a**) starting materials $MnFe_2O_4$@$Al(OH)_3$, **b**) $MnFe_2O_4$@HA, and **c**) $MnFe_2O_4$@HA-BP-PEG

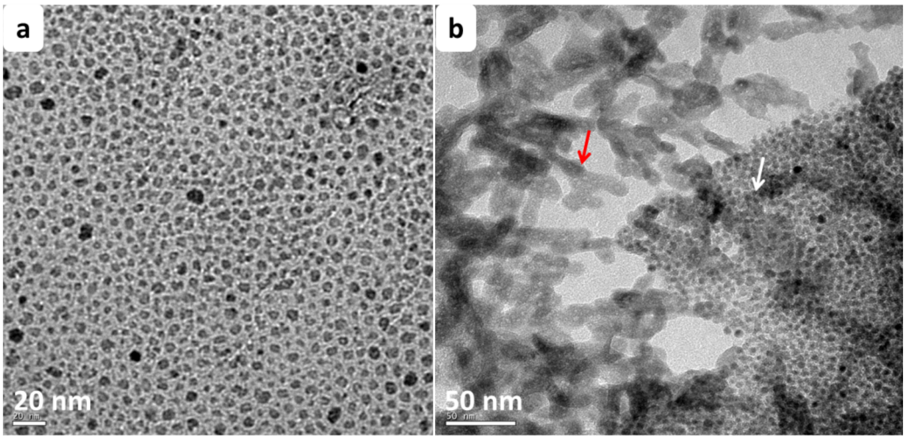

Figure S9. TEM images of **a**) precursors $MnFe_2O_4$ NPs and b) the product obtained by using $MnFe_2O_4$ NPs instead of $MnFe_2O_4$@$Al(OH)_3$ as precursors. The red arrow represents the hydroxyl apatite, and the white one represents the $MnFe_2O_4$ NPs.

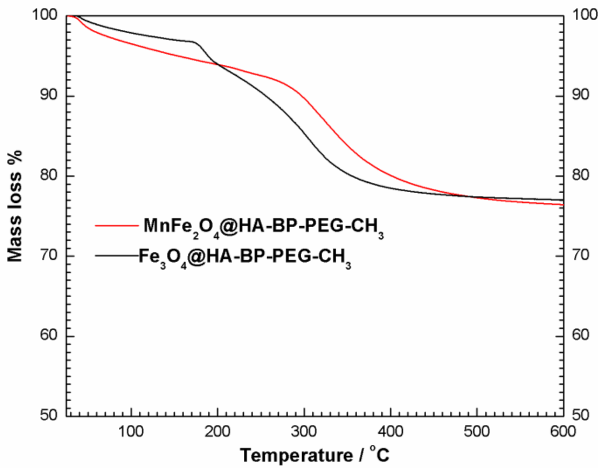

Figure S10.TGA of $MnFe_2O_4$@HA NPs and Fe_3O_4@HA

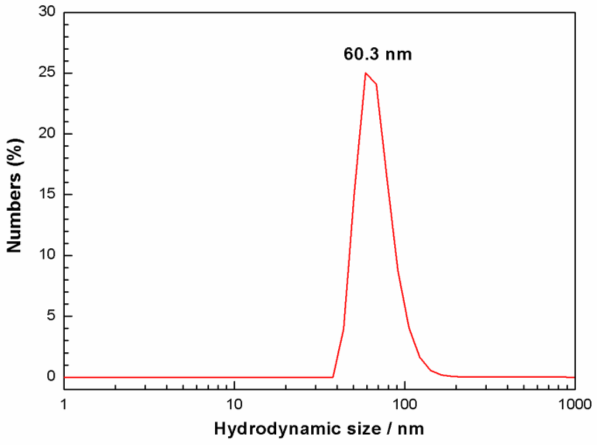

Figure S11. DLS size distribution of $MnFe_2O_4$@HA-BP-PEG-CH_3 NPs

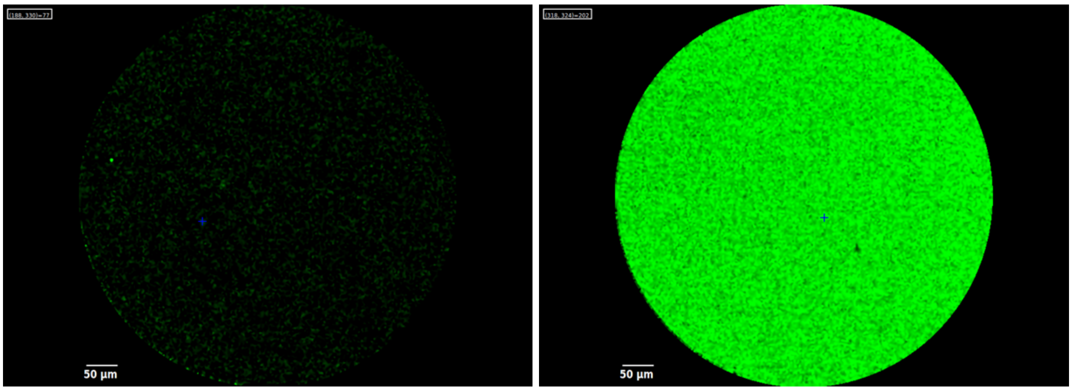

Figure S12. Fluorescent images of pure water (left, control) and the solution of Fe_3O_4@HA-BP-PEG-Me conjugates with Alexa Fluor® 488 (right). Excitation at 488 nm and emissions from 502 nm to 633 nm

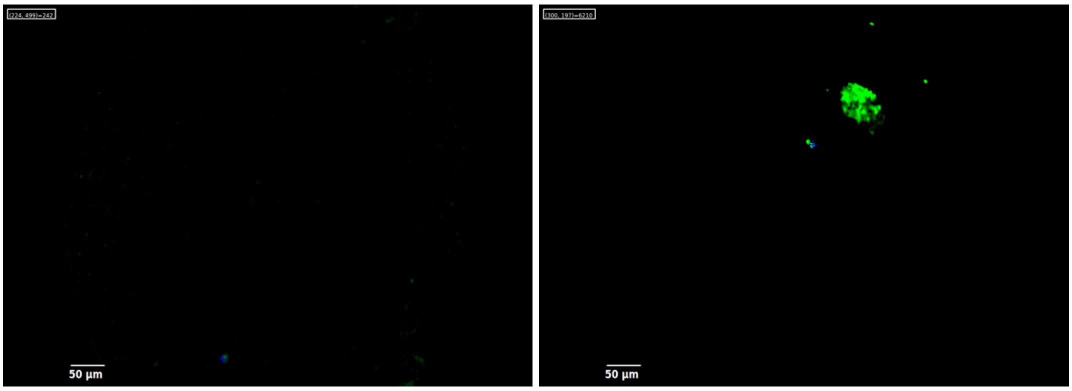

Figure S13. Fluorescent images of the tumour dissected from the control mice without NPs injected (left) and the mice intravenously administered RGD functionalised conjugates of Fe_3O_4@HA-BP-PEG-Me and Alexa Fluor® 488 (right, 18 hours post injection). Excitation at 488 nm and emissions from 502 nm to 633 nm. Images were acquired by a CellVizio DualBand confocal fluorescent endomicroscope using a S650B fiber optic microprobe. The 0.65 mm diameter fibre optic probe (3,3 micrometer lateral resolution) was inserted *in vivo* into 2 mm depth the tumour of a Nu/Nu female mouse harbouring a xenografted tumour of OVCAR ovarian carcinoma cell line (under ketamine/xylazine anaesthesia.).

1. X. Cui, S. Belo, D. Krüger, Y. Yan, R. T. M. de Rosales, M. Jauregui-Osoro, H. Ye, S. Su, D. Mathe, N. Kovács, I. Horváth, M. Semjeni, K. Sunassee, K. Szigeti, M. A. Green and P. J. Blower, *Biomaterials*, 2014, **35**, 5840-5846.
2. U. Blume, J. Orbell, M. Waltham, A. Smith, R. Razavi and T. Schaeffter, *Magnetic Resonance Materials in Physics Biology and Medicine*, 2009, **22**, 375-383.
3. L. I. Peng, *Chinese Journal of Pharmaceuticals*, 2009, **40**, 406-407.
4. R. T. M. de Rosales, R. Tavare, A. Glaria, G. Varma, A. Protti and P. J. Blower, *Bioconjugate Chemistry*, 2011, **22**, 455-465.
5. R. T. M. de Rosales, R. Tavare, R. L. Paul, M. Jauregui-Osoro, A. Protti, A. Glaria, G. Varma, I. Szanda and P. J. Blower, *Angewandte Chemie-International Edition*, 2011, **50**, 5509-5513.
